# Effective Anti-SARS-CoV-2 RNA Dependent RNA Polymerase Drugs Based on Docking Methods: The Case of Milbemycin, Ivermectin, and Baloxavir Marboxil

**Published:** 2020

**Authors:** Ali Hassan Daghir Janabi

**Affiliations:** Department of Veterinary Microbiology, College of Veterinary Medicine, University of Al-Qadisiyah, Diwaniyah City, Iraq

**Keywords:** Baloxavir, COVID-19, Ivermectin, Tadalafil

## Abstract

**Background:**

Severe Acute Respiratory Syndrome-coronavirus 2 (SARS-CoV-2) is a new virus with a global pandemic. Yet, no vaccine or efficient treatments are found against the disease. The viral RNA dependent RNA Polymerase (RdRP) is a suitable target for developing antiviral agents. SARS-CoV-2 RdRP was employed to test its binding activity with some drugs.

**Methods:**

Using some docking methods, RdRP was targeted by Milbemycins (MMs), Ivermectin (IMT), Baloxavir Marboxil (BM), and Tadalafil (TF), a phosphodiesterase type 5 inhibitor.

**Results:**

MM-A3 5-oxime (MMA35O), MM-A3 (MMA3), MM-A4 5-oxime (MMA45O), IMT, BM, and TF showed the highest binding affinity to RdRp.

**Conclusion:**

The drugs used in the present computational investigation are effective against the SARS-CoV-2 RdRP with high affinity values especially, milbemycins, ivermectin, and Baloxavir marboxil, which could further be studied in laboratory and clinical trials for saving millions of lives around the world.

## Introduction

A few drugs have been used for treating Coronavirus Disease 2019 (COVID-19) patients such as Remdesivir (RDS) and Favipiravir (FPV) which are considered as nucleotide and nucleoside, respectively, inhibitors that interfere with the assembly of the viral RNA. RDS is used for the treatment of Ebola ^1^ and Marburg viruses ^2^ and SARS and MERS infections ^3^. In a compassionate-clinical use of RDS in hospitalized COVID-19 patients breathing ambient air or on oxygen support, clinical improvement was seen in 36 of 53 patients (68%) ^4^ which is still a humble recovery especially in the case of more severe and critical patients. Like RDS, FPV (T-705; 6-fluoro-3-hydroxy-2-pyrazinecarboxamide) is a nucleoside analogue used as an antiviral drug that halts the replication of the viral RNA due to its incorporation in the newly-made RNA strand of wide-range viruses ^5^. FPV was recorded to induce inhibition to seasonal and avian influenza viruses even those strains that are resistant to neuraminidase inhibitors as approved using cell culture and experimental animals ^6^.

Due to the availability of the RdRP 3D structure ^7^, docking methods in the present work were employed to enhance production of drugs that bind with high affinities to the binding sites of the enzyme. This might block the activity of the enzyme, halt the viral RNA building, and eventually stop the viral replication.

## Materials and Methods

In the current study, drugs (Table 1) were employed to target active binding sites of the SARS-CoV-2 RdRP (PDB ID: 6m71; https://www.rcsb.org/), after removing cofactors from the structure using PyMOL ^8^. MMs, members of the macrocyclic lactone family, were selected according to a report that IMT, a member of the same family, was effective in minimizing the replication of the COVID-19 in cell culture ^9^. For BM, the selection was according to its inhibition effects on the polymerase-based initiation of mRNA of the influenza virus ^10^. In the case of TF selection, it was previously reported to prevent infection of a cell line by the Human Immunodeficiency Virus (HIV) ^11^. PyRx-Python Prescription (0.8) and Autodock vina plugin ^12^ were used for the prediction of the affinity values. In details, Standard Database Format (SDF) files of the ligands were downloaded from https://pubchem.ncbi.nlm.nih.gov/ and were converted into PDB file type *via* the use of BABEL plugin in the PyRX software. IMT was downloaded from the http://www.chemspider.com/ due to unavailability of the 3-dimensional structure in the https://pubchem.ncbi.nlm.nih.gov/. Then, both protein and ligand files were prepared for the docking process using PyRx software for removing waters, in which the PyRx software gets rid of water molecules automatically before generating pdbqt files. Grid boxes were optimized for free searching by the ligands on the whole enzyme molecule (Active and non-active sites). The dissociation constant (Kd) is predicted *via* auto dock built in the PyRx software that converts the affinity energy values (*kcal/mol*) into Kd according to the following equation:

DeltaG: Affinity energy (*kcal/mol*)
R: Gas constant
T: Room temperature (298.15°*K*=25°*C*)
Ki: Dissociation constant

PyMOL ^8^ was used to create the docking images. The active binding sites are located on motifs A to G; however, A and C motifs of the RdRP are considered important for the RNA template involvement. These sites are clamped by critical motifs F and G that ease the entry of this template *via* a groove to the active sites in the motifs A and C ^7^.

**Table 1.** Drugs utilized for the docking and their PubChem IDs

**PubChem CID**	**Drug**
**91617476**	Milbemycin A3 5-oxime
**9828343**	Milbemycin A3
**91617829**	Milbemycin A4 5-oxime
**ChemSpider ID7988461**	Ivermectin
**124081896**	Baloxavir marboxil
**110635**	Tadalafil

Interactions (2 and 3D models) between the RdRP and the ligands were visualized using Discovery Studio Visualizer v20.1.0.19295 (BIOVIA). The images were generated using Discovery Studio Visualizer v20.1.0. 19295 (BIOVIA), Microsoft PowerPoint, and GIMP v2.10.18.

## Results

**Figure 1.**
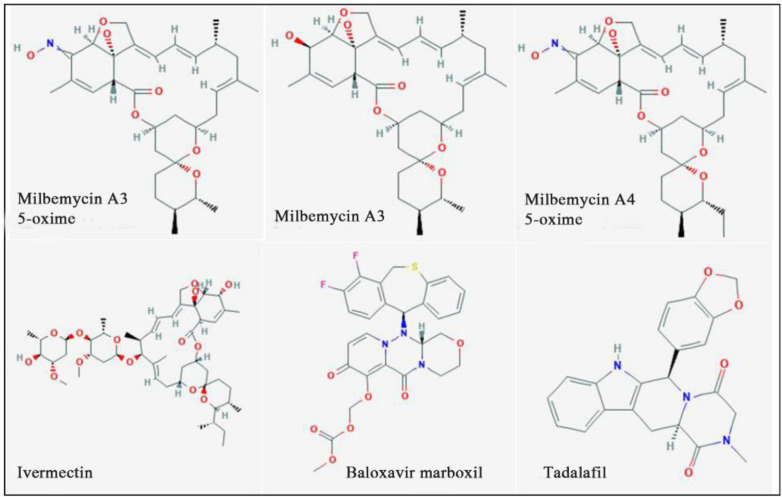
Chemical structures of the study ligands.

The outcomes of the present investigation shown in table 2 unveiled the highest affinity values for MMA-35O, MMA3, MMA45O, and IMT when binding to RdRp followed by BM and TF. The chemical structures of these ligands are displayed in figure 1.

**Table 2.** Autodock Vina binding affinity values of drugs to the COVID-19 RdRp

**Drugs**	**Binding affinity values (*kcal/mol*)**
**Milbemycin A3 5-oxime**	−10.1
**Milbemycin A3**	−10.1
**Milbemycin A4 5-oxime**	−9.2
**Ivermectin**	−9.2
**Baloxavir marboxil**	−9.2
**Tadalafil**	−8.9

**Figure 2.**
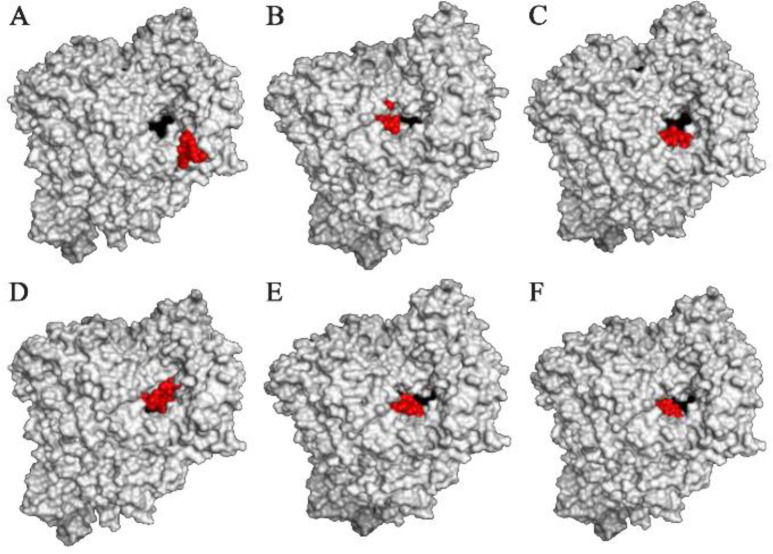
RNA dependent RNA polymerase (grey, surface-hydrophobicity mode) of the SARS-CoV-2 (COVID-19) in complex with different ligand drugs (red); A) MMA35O binds to the B motif; B) MMA3 binds to the F motif; C) MMA45O binds to the B and F motifs; D) IMT binds to the C and F motifs; E) BM binds to the B and F motifs; F) TF binds to the B and F motifs.

**Figure 3.**
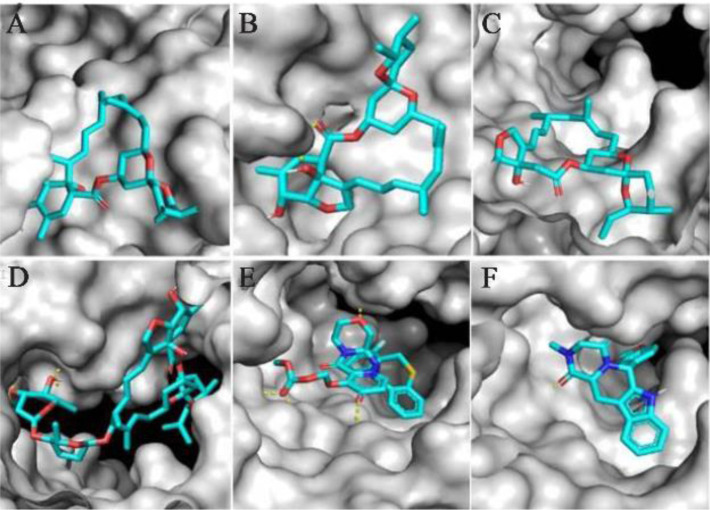
Zoom-in images of the RNA dependent RNA polymerase (grey) of the SARS-CoV-2 (COVID-19) with different ligand drugs (cyan); A) MMA35O binds to the B motif; B) MMA3 binds to the F motif; C) MMA45O binds to the B and F motifs; D) IMT binds to the C and F motifs; E) BM binds to the B and F motifs; F) TF binds to the B and F motifs.

**Figure 4.**
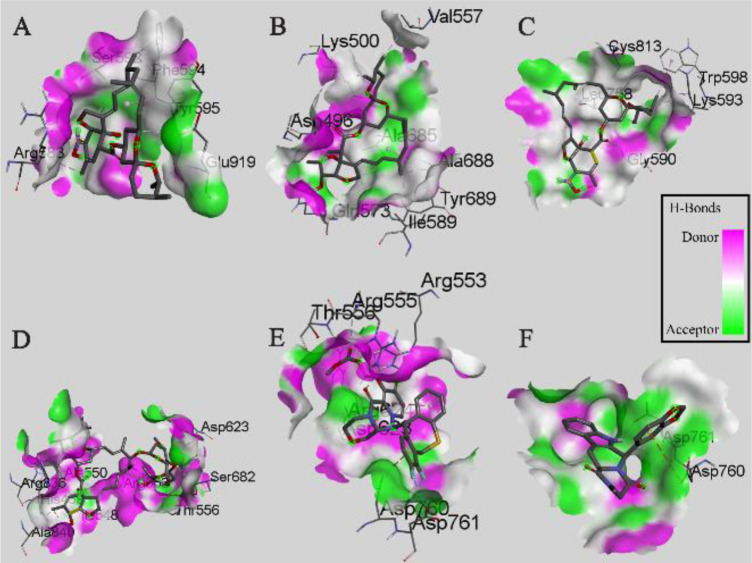
Interactions (3D-model) of ligands with the RNA dependent RNA polymerase of the SARS-CoV-2 (COVID-19) based on hydrogen bond style visualized by Discovery Studio Visualizer v20.1.0. 19295; A) MMA35O; B) MMA3; C) MMA45O; D) IMT; E) BM; F) TF.

**Figure 5.**
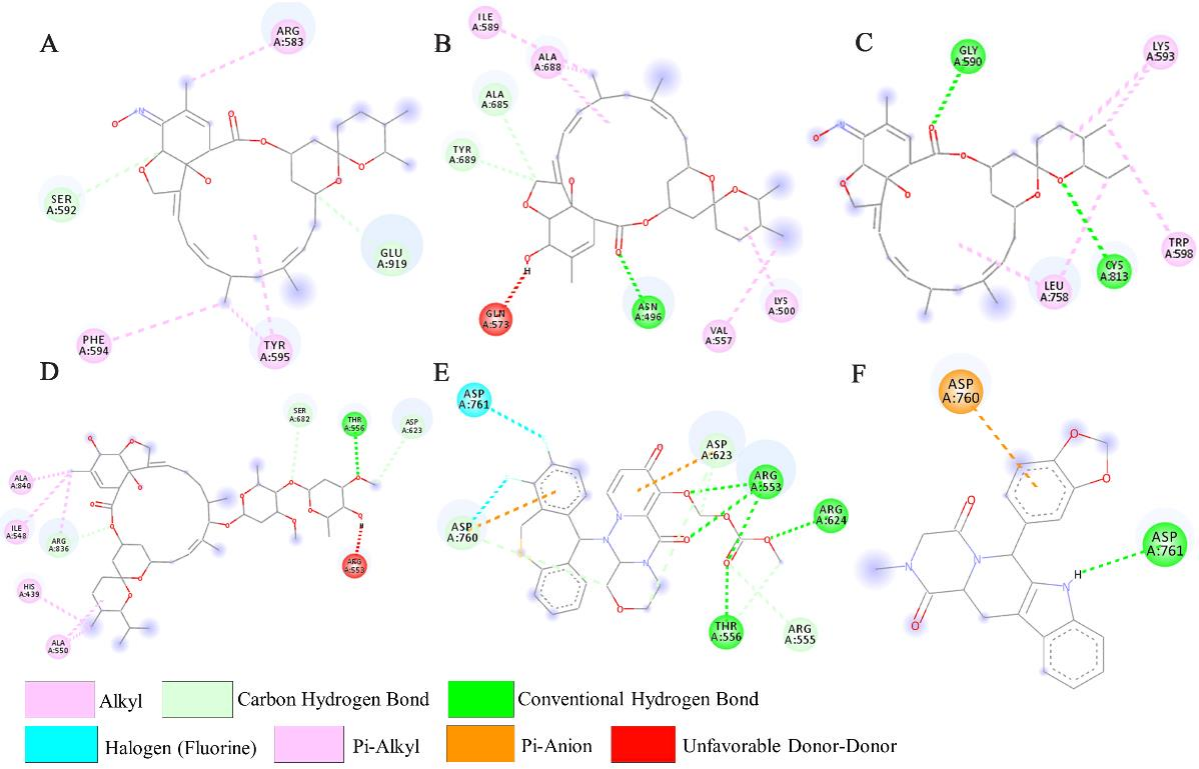
Interactions (2D-model) of ligands with the RNA dependent RNA polymerase of the SARS-CoV-2 (COVID-19) based on hydrogen bond style visualized by Discovery Studio Visualizer v20.1.0.19295; A) MMA35O; B) MMA3; C) MMA45O; D) IMT; E) BM; F) TF.

The results showed that the palm subdomain in motif B of the RdRP is engaged by the MMA35O (Figure 2A). The bonds of the attachment between the ligand and the protein are shown in figures 3–5A. MMA3 revealed its binding to the bindings sites in the RNA template tunnel groove of the F motif of the RdRP enzyme (Figure 2B). The linkages between the ligand and the enzyme are displayed in figures 3–5B. Moreover, MMA45O displayed attachment to the binding sites located in the B and F motifs in the way of RNA template entry (Figure 2C). The ligand-protein based bonds are shown in figures 3–5C. IMT revealed binding to the binding sites present in the C and F motifs in the inlets of RNA template and NTPs and the outlet of the product hybrid (Figure 2D). For the linkages, the ligand-enzyme connection is shown in figures 3–5D. For the BM, the binding site location was in the B and F motifs of the inlets of RNA template and NTPs and the outlet of the product hybrid (Figure 2E). The adherence of the ligand to the protein is displayed in figures 3–5E. Finally, TF provided binding properties at the same location of that from BM (Figure 2F). The ligand-enzyme bonds are shown in figures 3–5F.

## Discussion

The active sites of the RdRP, also known as nsp12, are located in the motifs A to G ^7^. This activity is probably due to different directions taken by the major components; RNA template, NTPs, and product hybrid, involved in the building of the new viral RNA strands. The finding of the present study revealed that the palm subdomain in the motif B of the RdRP is bound by the MMA35O. The palm subdomain (Residues T582-P620 and T680-Q815) is an important part of the core region of the RdRP that participates in the stability of the enzyme structure along with fingers subdomain (Residues L366-A581 and K621-G679) and a thumb subdomain (Residues H816-E920) ^7^. Hypothetically, the presence of the MMA35O in the bound region may increase the chances of halting the enzyme activity especially when this region is considered adjacent to the passage of the RNA building materials. MMA3 showed its binding to the F motif of the RdRP enzyme. The F motif is a critical part through which the RNA template is expected to enter to the active sites in the motifs A and C to engage in the process of the RNA replication. This attachment region is located in the active groove that is used as an RNA template entry passage. Typically, this means that the occurrence of the ligand in this pocket might enhance the chance of disrupting the replication of the viral RNA strand.

Another molecule that expressed binding to RdRP is MMA45O. The drug attached to the binding sites in the motifs B and F. The bound ligand was seen sitting in the groove, RNA template passage entry to the motif A and C, clamped by the F motif. Such occasion might interfere with the activity of the RdRP, thereby stopping its activity and eventually finishing the cycle of viral replication. Motif A (Residues 611-TPHLMGW-DYPKCDRAM-626) has a conserved divalent-cation-binding residue (D618) which is similar to that in other viral RdRP such as in HCV (D220) and poliovirus (D233). IMT revealed binding to motif C and F. Motif C consists of residues of 753-FSMMILSDDAVVCFN-767 including the catalytic residues of 759-SDD-761 ^7^.

In the present study, Baloxavir marboxil displayed successful binding to the active sites in the B and F motifs of the RdRP. Baloxavir marboxil suppresses the activity of the viral replication of the influenza virus by inhibiting the polymerase PA subunit ^13^. Since the target of this drug is the viral polymerase, this might be a great drug of choice for treating COVID-19 patients. The TF showed strong binding activity to the polymerase of the virus. Tadalafil is an inhibitor to the phosphodiesterase type 5 component utilized to manage erectile dysfunction ^14^. This could be applied as a great drug of choice for treating COVID-19 cases especially when this drug is used to treat pulmonary arterial hypertension ^15^.

## Conclusion

The current work provided a group of drugs that could be further studied *in vitro*, *in vivo*, and in clinical trials to find whether they are effective against the SARS-CoV-2 (COVID-19) virus and thereby, quickly helping to manage the pandemic around the world.
